# Biological Activity of Volatiles from Marine and Terrestrial Bacteria

**DOI:** 10.3390/md8122976

**Published:** 2010-12-22

**Authors:** Stefan Schulz, Jeroen S. Dickschat, Brigitte Kunze, Irene Wagner-Dobler, Randi Diestel, Florenz Sasse

**Affiliations:** 1 Institute of Organic Chemistry, University of Braunschweig—Institute of Technology, Hagenring 30, 38106 Braunschweig, Germany; E-Mail: j.dickschat@tu-bs.de (J.S.D.); 2 Research Group Microbial Communication, Helmholtz Center for Infection Research, Inhoffenstrasse 7, 38124 Braunschweig, Germany; E-Mails: brigitte.kunze@helmholtz-hzi.de (B.K.); irene.wagner-doebler@helmholtz-hzi.de (I.W.-D.); 3 Department of Chemical Biology, Helmholtz Center for Infection Research, Inhoffenstrasse 7, 38124 Braunschweig, Germany; E-Mails: randi.diestel@helmholtz-hzi.de (R.D.); florenz.sasse@helmholtz-hzi.de (F.S.)

**Keywords:** lactones, sulfur compounds, pyrazines, bacterial communication, quorum-sensing, ketones, aromatic compounds, agar diffusion assay, terpenes

## Abstract

The antiproliferative activity of 52 volatile compounds released from bacteria was investigated in agar diffusion assays against medically important microorganisms and mouse fibroblasts. Furthermore, the activity of these compounds to interfere with the quorum-sensing-systems was tested with two different reporter strains. While some of the compounds specific to certain bacteria showed some activity in the antiproliferative assay, the compounds common to many bacteria were mostly inactive. In contrast, some of these compounds were active in the quorum-sensing-tests. *γ*-Lactones showed a broad reactivity, while pyrazines seem to have only low intrinsic activity. A general discussion on the ecological importance of these findings is given.

## 1. Introduction

Marine bacteria are a rich source of natural products with interesting biological and pharmacological activities [1,2]. While most research on marine bacterial metabolites focusses on compounds with moderate polarity isolable from fermentation experiments, relatively few volatile compounds from marine as well as other bacteria are known [3]. Many bacteria, marine or terrestrial, produce volatiles, but the specific function of these compounds is known only in few cases. It seems likely that the volatiles are used either in intra- or inter-specific communication and/or in the chemical defence against other microorganisms. Several studies have shown that bacterial volatiles can indeed influence the growth of other organisms [4]. Bacterial volatiles can impact, most often inhibit, the growth of fungi [4–9], but only few studies describe the activities of certain compounds. Compounds identified from fungistatic soil bacteria inhibiting spore germination of certain fungi were acetamide, trimethylamine, 3-methyl-2-pentanone, benzaldehyde, *N*,*N*-dimethyloctylamine, benzothiazole, 1-butanamine, methanamine, phenylacetaldehyde, and 1-decene [10,11]. Benzothiazole, cyclohexanol, nonanal, decanal, dimethyl trisulfide, and 2-ethyl-1-hexanol, the latter most likely an anthropogenic artefact, identified from several *Pseudomonas* strains, were active in inhibiting growth of the plant pathogen, *Sclerotinia sclerotiorum* [12]. 1-Undecene and dimethyl disulfide (**2**), released by several bacteria, were also active [4], despite the fact that the latter proved to be inactive in other studies [10–12]. Effects on plants have been described for 2,3-butanediol (**47**) and acetoin (**48**) which improve plant growth in *Arabidopsis thaliana*, and also effects on animals have been described [4]. On the other hand, reports on interbacterial effects of volatile compounds are rare. Indole acts as a signalling compound in *Escherichia coli* [13] and shows effects on other bacteria [14] while volatile carboxylic acids inhibit spore formation in several pathogenic bacteria [15].

More or less all studies performed so far concentrated on the evaluation of the effects of readily available compounds. While these studies showed activity in some cases, questions remain about the activity potential of other components. The volatiles identified so far can be roughly divided into different groups depending on their occurrence. While there are common compounds produced by many different, often unrelated bacteria, e.g., dimethyl disulfide (**52**), or at least by certain groups as geosmin (**49**) and 2-methylisoborneol (**50**) found in actinobacteria, myxobacteria, and cyanobacteria, some are unique for certain strains as the lactones **4**–**11** [3].

In the present study 52 compounds were tested for their biological effects, covering strain or species specific compounds (**1**–**44**) and commonly occurring ones (**45**–**52**). In a first set of experiments we established a general profile of the biological activities of these compounds. Therefore, the antiproliferative activity with bacteria, fungi, and murine fibroblasts was tested. The antimicrobial assays were focussed on medically important strains. In a second set of experiments the possibility of interference with well known bacterial chemical communication channels was probed. In this context, the compounds were investigated in an assay on bacterial crosstalk [16] to demonstrate whether they can influence the known bacterial quorum-sensing signalling pathway via *N*-acylhomoserine lactones (AHLs).

**Figure 1:**
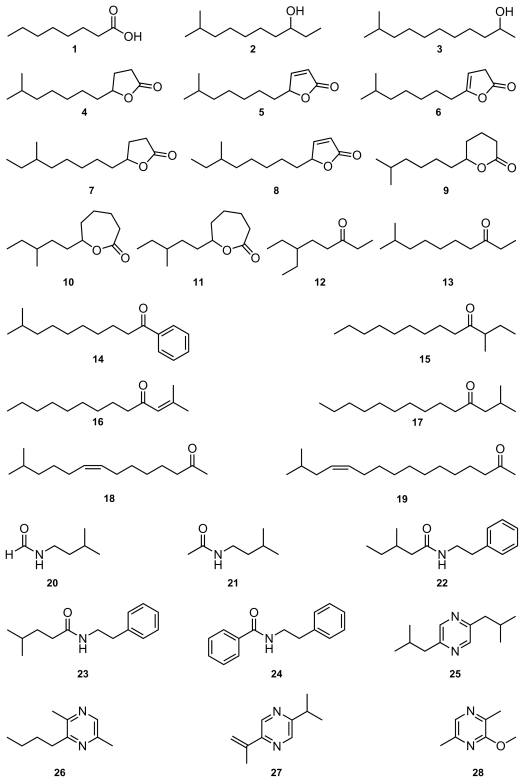

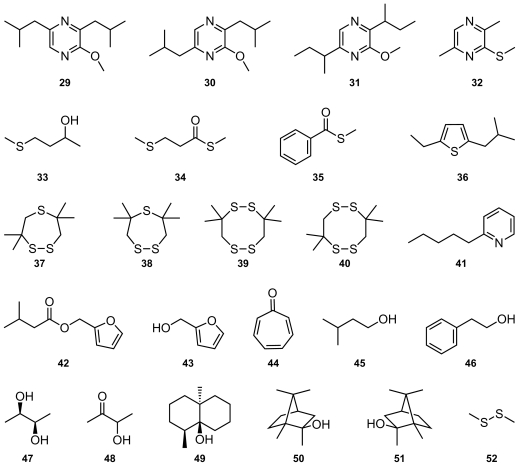
Compounds used in this study. Acids, alcohols, lactones, ketones, amides, pyrazines, sulfur compounds, aromatic compounds, and substances commonly occurring as bacterial volatiles (**45**–**52**).

The compounds tested (Figure 1) were identified from different marine bacteria (see Table 1 for references). Several compounds originally known from terrestrial bacteria are also included to compare activities between compounds originating from terrestrial or marine sources. Nevertheless, there is no clear cut difference in chemistry between terrestrial and bacterial microorganisms. Common compounds usually occur in both areas.

## 2. Results and Discussion

Classical agar diffusion assays were performed using the fungi *Aspergillus fumigatus*, *Botrytis cinerea*, and *Pythium debaryanum* as well as the yeasts *Hansenula anomala*, *Saccharomyces cerevisiae*, and *Candida albicans*. Furthermore, the bacteria *Pseudomonas aeruginosa*, *Klebsellia pneumoniae*, *Staphylococcus aureus*, *Micrococcus luteus*, *Mycobacterium phlei* and a mutant of *Escherichia coli* which is defective in the *tolC* gene [17] were used as test organisms. The results are shown in Table 1. Octanoic acid (**1**) is active against hyphal fungi and yeasts, as are the amides **22** and **23**. *γ*-Butyrolactones **4**–**8** are active against fungi, yeasts, and bacteria, with a large influence of a double bond in the ring. Expansion of the ring to *δ*- (**9**) or *∈*-lactones (**10** and **11**) reduces the activity. 9-Methyl-3-decanol (**2**), the characteristic component of the headspace volatiles of *Myxococcus xanthus*, shows activity against Gram-positive bacteria and the *tolC* mutant of *Escherichia coli*, while alcohol **3** only inhibits the sensitive *E. coli* mutant. Pyrazines (**25**–**32**) as well as ketones (**12**–**19**) are largely inactive with the exception of (*Z*)-15-methylhexadec-12-en-2-one (**19**), showing broad activity. *S*-Methyl benzothioate (**35**) is the only sulfur containing compound with activity against fungi and yeast, while all others (**32**–**40**) are inactive. 2-Pentylpyridine (**41**), produced by a *Streptomyces* strain [18], is slightly active against *S. cerevisiae*, *E. coli*, and *K. pneumoniae*. In contrast, the latter bacterium and *C. albicans* are the only microorganisms furfuryl isovalerate (**42**) is not active against.

The results discussed so far were obtained with compounds produced only by specific bacterial strains. In contrast, the compounds common to many bacteria showed no inhibitory activity. This is even true for components as dimethyl disulfide (**52**) which showed activity in other assays [4].

Additionally, the cytotoxic activity against L-929 mouse fibroblasts was investigated in concentration dependant assays. The obtained minimal inhibition concentration (MIC) values indicated moderate or no activity. The most active compounds were furfuryl isovalerate (**42**, MIC 62 *μ*mol/L), the ketone **19** (MIC 73 *μ*mol/L), the lactones **10** and **11** (MIC 260 and 280 *μ*mol/L), as well as the pyrazine **28** (MIC 320 *μ*mol/L). All these compounds were also active in at least some antimicrobial assays except **28**.

All compounds were also investigated for their activity in *N*-acylhomoserine lactone (AHL) mediated bacterial communication systems (quorum-sensing). These test were performed using green fluorescent protein (gfp) equipped reporter strains capable of sensing AHLs with certain side chain specificity. The used *E. coli* MT102 (pJBA132) reporter shows the highest sensitivity for *N*-(3-oxohexanoyl)homoserine lactone (3-oxo-C6-AHL) [32], while the *Pseudomonas putida* F117 (pKR-C12) reporter is very sensitive to *N*-(dodecanoyl)homoserine lactone (C12-AHL) [33]. The corresponding AHLs were added to the sensor strains together with the test compound and the resulting reduction or increase of fluorescence was recorded (Table 2). Values of more than 50% were considered significant.

Several compounds were able to modify the sensor response. The lactones **5**–**9** inhibited the response of the C12-AHL sensor. Especially the *δ*-lactone **9** was highly active. In contrast, the 3-oxo-C6-AHL sensor showed a different behavior. In particular **4** and **7** stimulated this sensor. This influence may be due to the structural similarity between the lactones and the AHLs. Ketones also proved to be active, with **15**, **16**, **18**, **19**, and the related alcohol **3** reducing activity of the C12-AHL sensor, while **17** did so with the 3-oxo-C6-AHL sensor, as did the pyridine derivative **41**. The sulfur containing ester **34** and furfuryl isovalerate (**42**), also one of the most active compounds in the antimicrobial assays, were the only compounds able to reduce the activity of both sensors. In this assay also compounds common to many different bacteria showed some activity, reducing the effectivity of the 3-oxo-C6-AHL sensor. These compounds were the alcohols **45** and **46**, and both enantiomers of methylisoborneol (**50** and **51**). In summary, 25% of the compounds were able to reduce activity of the C12-AHL sensor, while 19% show inhibitory activity against 3-oxo-C6-AHL and 13% enhanced its activity.

The concentration of the compounds used in the sensor tests was quite high and dilution experiments usually led to reduced activity (see Table 2). The concentration of compounds released by bacteria can be quite high close to the bacterial membrane, their point of release. Therefore, nearby other bacteria might well experience relatively high concentrations of such metabolites, pointing to the possibility that these compounds might have ecological relevance for known bacterial communication channels.

In summary, the results show that bacterial volatiles can, despite their relatively small size, influence different biological traits. Often it is not clear why bacteria produce volatiles. One interesting observation is that some special compounds produced by only few organisms can have antimicrobial activity and therefore might ensure survival of the bacterium when under competition with other bacteria. Contrary to polar and larger compounds they are transported by air and are useful for bacteria under certain conditions, but can also travel in water with high velocity because of their small size. The volatiles can also interfere with bacterial communication channels and are ideally suited for being signalling compounds by themselves. Their potential receptors are not known because only few bacterial communication mechanisms have been established so far (see Introduction). Finally, in some cases they may also serve as a means of detoxification, as recently proposed for some pyrazines[34]. Interestingly, the pyrazines show almost no antimicrobial activity. The activity of the volatiles against proliferative cells can be regarded as quite low with the exception of furfuryl isovalerate, a surprisingly active compound in several aspects.

Finally, there is no clear difference in activity between volatiles produced by marine or terrestrial bacteria. The current known origin of the these compounds from bacteria either of terrestrial or marine habitats is probably pure chance. Most likely terrestrial strains will be found in the future that produce compounds only known from marine sources so far, e.g., sulfur compounds **37**–**40**, and *vice versa*.

In summary, we have shown that bacterial volatiles can have a multitude of activities. Their production has to be taken into account in future studies, especially when researching aspects of the bacterial chemical communication systems and their chemical defence.

## 3. Experimental Section

### 3.1. Antimicrobial assay

Antimicrobial activities were determined by agar diffusion assays using paper discs of 6 mm diameter soaked with 20 *μ*L of methanolic solution of the test compound. Microorganisms from the HZI collection were grown on standard media and seeded into liquid agar medium to a final O.D. of 0.01 (bacteria) or 0.1 (yeasts). Spores of fungi were seeded according to experience. Plates were incubated at 30 °C and the diameter of resulting inhibition zones were measured after 1 or 2 days.

### 3.2. Cell proliferation assay

L-929 mouse fibroblasts were obtained from the German Collection of Microorganisms and Cell Cultures (DSMZ) and cultivated at 37 °C and 10% CO_2_ in DME medium (high glucose) supplemented with 10% fetal calf serum. Inhibition of proliferation was measured in microtiter plates. 60 *μ*L of serial dilutions of the test compounds were given to 120 *μ*L of the suspended cells (50,000/mL) in wells of 96-well plates. After 5 days growth each well was judged visually under the microscope. The lowest concentration of a compound that led to a clearly reduced growth is given as minimal inhibitory concentration (MIC).

### 3.3. Acylhomoserine lactone sensors

The AHL biosensor strains *E. coli* MT102 (pJBA132) and *Pseudomonas putida* F117 (pRK-C12) were grown in Luria-Bertani (LB) medium with 25 *μ*g/mL tetracycline at 37 °C and with 20 *μ*g/mL gentamycin at 30 °C, respectively. Overnight cultures (50 mL) of the strains were diluted with the same volume of fresh medium, incubated for about one more hour and adjusted to an OD620 of 1.0. Aliquots (100 *μ*L) of these cultures were transferred by pipet into 96-well microtiter plates which contained 90 *μ*L of LB medium, and 5 *μ*L of the test compounds in the concentrations reported in Table 2. To test the compounds for inhibitory activity on quorum-sensing, the sample wells contained 5 *μ*L of the corresponding AHL *N*-3-oxohexanoylhomoserine lactone for *E. coli* MT102, *N*-dodecanoylhomoserine lactone for *P. putida* F117) at a concentration of 0.25 *μ*g/mL. Samples containing only the corresponding AHL without test compound were used as positive control and samples containing solvent (10 *μ*L) alone were used as negative control. The microtiter plates were incubated at 30 °C with shaking. After 0, 4, 8, and 24 h growth at an OD620 nm, fluorescence (excitation wavelength 485 nm, detection wavelength of 535 nm) was determined in a Victor 1420 Multilabel Counter (Perkin Elmer). Fold induction of fluorescence in the test samples was calculated by dividing their specific fluorescence (gfp535/OD620) by the specific fluorescence of the negative control. Relative activity was calculated by comparing the fold induction of positive control samples (only homoserine lactones) to samples containing samples with test compounds and the corresponding homoserine lactone.

## Figures and Tables

**Table 1:** Agar diffusion inhibition assay. Asf: *Aspergillus fumigatus*; Boc: *Botrytis cinerea*; Caa: *Candida albicans*; Pyd: *Pythium debaryanum*; Hna: *Hansenula anomala*; Scc: *Saccharomyces cerevisiae*; tolC: *E. coli tolC* mutant; Psa: *Pseudomonas aeruginosa*; Kbp: *Klebsellia pneumoniae*; Sta: *Staphylococcus aureus*; Mcl: *Micrococcus luteus*; Myp: *Mycobacterium phlei*; Amount: amount of compound used in the agar diffusion assay; mm: diameter of inhibition zone; i: incomplete inhibition; MIC: cytotoxic activity against L-929 mouse fibroblasts, minimal inhibition concentration; Oc: source.

No	Compound	Amount	Asf	Boc	Pyd	Caa	Hna	Scc	tolC	Psa	Kbp	Sta	Mcl	Myp	MIC	Oc
		*μ*g	mm	mm	mm	mm	mm	mm	mm	mm	mm	mm	mm	mm	*μ*mol	
1	Octanoic acid	112	10i	20		11i		11i	0	0	0	0	0	0	480	(1)
2	9-Methyl-3-decanol	156	0	0		0		0	12	0	0	8	13	20	560	[19]
3	10-Methylundecan-2-ol	382	0	0		0		0	8	0	0	0	0	0	420	[20]
4	10-Methylundecan-4-olide	416	11	14		0		0	14	0	0	8i	10i	19	430	[21]
5	10-Methylundec-2-en-4-olide	346	15	14		9i		14	17	11i	0	9	8i	11	363	[21]
6	10-Methylundec-3-en-4-olide	218	9i	9		0		10	13	13i	0	8i	8i	12	230	[21]
7	10-Methyldodecan-4-olide	428	8i	10i		0		0	15	12i	0	8i	8i	13	415	[21]
8	10-Methyldodec-2-en-4-olide	140	8i	8		0		9	20	0	0	8	8	10	135	[21]
9	10-Methylundecan-5-olide	250	0	0		0		0	0	0	0	0	0	0	780	[21]
10	9-Methylundecan-6-olide	28	0	0		0		0	8i	0	0	0	0	18i	260	[22]
11	10-Methylundecan-6-olide	30	0	0		0		0	0	0	0	0	0	0	280	[22]
12	6-Ethyl-3-octanone	304	0	0		0		0	0	0	0	0	0	0	*>*1000	(2)
13	9-Methyl-3-decanone	162	0	0		0		0	9	0	0	0	0	0	590	[19]
14	9-Methyl-1-phenyldecan-1-one	200	0	0	0		0	0	0	0	0	0	0	0	500	[20]
15	3-Methyltridecan-4-one	528	0	0		0		0	0	0	0	0	0	0	510	[20]
16	2-Methyltridec-2-en-4-one	414	0	0		0		8i	0	0	0	0	0	0	405	[20]
17	2-Methyl-4-tetradecanone	488	0	0		0		0	0	0	0	0	0	0	443	[20]
18	(*Z*)-13-Methyltetradec-8-en-2-one	280	0	0		0		0	0	0	0	0	0	0	257	[23]
19	(*Z*)-15-Methylhexadec-12-en-2-one	280	8i	9i		0		9	9	12i	0	13i	10	13	73	[23]
20	Isopentyl formamide	524	0	0		0		0	0	0	0	0	0	0	*>*1000	[20]
21	Isopentyl acetamide	386	0	0		0		0	0	0	0	0	0	0	*>*1000	[20]
22	3-Methyl-*N*-(2-phenylethyl)pentanamide	290	12i	13i		15i		0	11	0	0	0	0	0	815	[24]
23	4-Methyl-*N*-(2-phenylethyl)pentanamide	130	10i	0		10i		0	10i	0	0	0	0	0	365	[24]
24	*N*-(2-phenylethyl)benzamide	282	0	0		0		0	0	0	0	0	0	0	773	[24]
25	2,5-Diisobutylpyrazine	1322	0	0		0		0	20	0	0	0	0	0	*>*1000	[25]
26	2-Butyl-3,6-dimethylpyrazine	644	0	0		0		0	10	0	0	0	0	0	*>*1000	[23]
27	2-Isopropenyl-5-isopropylpyrazine	178	0	0		0		0	0	0	0	0	0	0	680	[26]
28	2-Methoxy-3,6-dimethylpyrazine	217	0	0		0		0	0	0	0	0	0	0	320	[26]
29	2-Methoxy-3,5-diisobutylpyrazine	354	0	0		0		0	10	0	0	0	0	0	*>*1000	[26]
30	2-Methoxy-3,6-diisobutylpyrazine	354	0	0		0		0	9	0	0	0	0	0	327	[26]
31	2-Methoxy-3,6-di-sec-butylpyrazine	106	0	0		0		0	0	0	0	0	0	0	487	[26]
32	2-Methylsulfanyl-3,6-dimethylpyrazine	916	0	0		0		0	0	0	0	0	0	0	425	[26]
33	4-Methylsulfanyl-2-butanol	220	0	0		0		0	0	0	0	0	0	0	*>*1000	[23]
34	*S*-Methyl 3-(Methylsulfanyl)propanethioate	1100	0	0		0		0	0	0	0	0	0	0	*>*1000	[27]
35	*S*-Methyl benzothioate	406	10i	11i		10i		0	8i	0	0	0	0	0	*>*1000	[28]
36	2-Ethyl-5-isobutylthiophene	238	0	0		0		0	0	0	0	0	0	0	*>*1000	(2)
37	3,3,6,6-Tetramethyl-1,2,5-trithiepane	200	0	0	0		0	0	0	0	0	0	0	0	705	[29]
38	3,3,7,7-Tetramethyl-1,2,5-trithiepane	200	0	0	0		0	0	0	0	0	0	0	0	*>*1000	[29]
39	3,3,8,8-Tetramethyl-1,2,5,6-tetrathiocane	200	0	0	0		0	0	0	0	0	0	0	0	*>*1000	[29]
40	3,3,7,7-Tetramethyl-1,2,5,6-tetrathiocane	200	0	0	0		0	0	0	0	0	0	0	0	*>*1000	[29]
41	2-Pentylpyridine	1198	0	0		0		8i	8	0	8i	0	0	0	*>*1000	(3)
42	Furfuryl isovalerate	494	11i	15		0		12	8	13	0	13	9	12	62	[26]
43	2-Furanmethanol	200	0	0	0		0	0	0	0	0	0	0	0	*>*1000	[30]
44	Tropone	200	0	0	0		0	0	0	0	0	0	0	0	*>*1000	[27,28]
	**Common Compounds**															
45	3-Methylbutanol	200	0	0	0		0	0	0	0	0	0	0	0	*>*1000	[3]
46	2-Phenylethanol	200	0	0	0		0	0	0	0	0	0	0	0	*>*1000	[21,23,27]
47	(2*R*,3*R*)-Butanediol	200	0	0	0		0	0	0	0	0	0	0	0	*>*1000	[3]
48	Acetoin	200	0	0	0		0	0	0	0	0	0	0	0	*>*1000	[3]
49	Geosmin	200	0	0	0		0	0	0	0	0	0	0	0	*>*1000	[21]
50	(+)-*R*-Methylisoborneol	200	0	0	0		0	0	0	0	0	0	0	0	*>*1000	[21]
51	(−)-*S*-Methylisoborneol	200	0	0	0		0	0	0	0	0	0	0	0	240	
52	Dimethyl disulfide	200	0	0	0		0	0	0	0	0	0	0	0	*>*1000	[21]

(1) Occurs in unclassified myxobacteria [31].

(2) Occurs in *Myxococcus xanthus* DK 1622 [31].

(3) Occurs in *Streptomyces* sp. F5 [18].

**Table 2:** Acylhomoserine lactone assay using *N*-3-oxohexanoylhomoserine lactone for *E. coli* MT102 (C_6_-AHL) and *N*-dodecanoylhomoserine lactone for *P. putida* F117 (C_12_-AHL). The maximum reduction relative to the native ligand is reported (%). Negative values indicate no inhibition, but increase of the fluorescence induction. Here the maximum induction rate relative to the native ligand (100%) is given.

No	Compound	Conc.	C_12_-AHL	C_6_-AHL
		*μ*g/mL	%	%
1	Octanoic acid	140	23	15
2	9-Methyl-3-decanol	250	6	−198
3	10-Methylundecan-2-ol	477	60	3
4	10-Methylundecan-4-olide	500	7	−550
5	10-Methylundec-2-en-4-olide	432	59	−40
6	10-Methylundec-3-en-4-olide	272	52	−16
7	10-Methyldodecan-4-olide	535	71	−70
8	10-Methyldodec-2-en-4-olide	175	61	−32
9	10-Methylundecan-5-olide	312	82	15
10	9-Methylundecan-6-olide	35	20	12
11	10-Methylundecan-6-olide	37	26	41
12	6-Ethyl-3-octanone	250	−15	−39
13	9-Methyl-3-decanone	250	10	−115
14	9-Methyl-1-phenyldecan-1-one	500	1	−27
15	3-Methyltridecan-4-one	660	59	−3
16	2-Methyltridec-2-en-4-one	517	52	−9
17	2-Methyl-4-tetradecanone	610	46	53
18	(*Z*)-13-Methyltetradec-8-en-2-one	315	56	−18
19	(*Z*)-15-Methylhexadec-12-en-2-one	315	82	17
20	Isopentyl formamide	655	26	34
21	Isopentyl acetamide	482	15	31
22	3-Methyl-*N*-(2-phenylethyl)pentanamide	362	12	18
23	4-Methyl-*N*-(2-phenylethyl)pentanamide	162	−13	19
24	*N*-(2-phenylethyl)benzamide	350	28	49
25	2,5-Diisobutylpyrazine	500	−5	−94
26	2-Butyl-3,6-dimethylpyrazine	500	13	40
27	2-Isopropenyl-5-isopropylpyrazine	222	35	72
28	2-Methoxy-3,6-dimethylpyrazine	500	40	60
29	2-Methoxy-3,5-diisobutylpyrazine	250	10	−92
30	2-Methoxy-3,6-diisobutylpyrazine	500	26	−102
31	2-Methoxy-3,6-di-sec-butylpyrazine	132	33	36
32	2-Methylsulfanyl-3,6-dimethylpyrazine	500	5	31
33	4-Methylsulfanyl-2-butanol	275	26	32
34	*S*-Methyl 3-(Methylsulfanyl)propanethioate	1375	54	85
		275		40
35	*S*-Methyl benzothioate	500	−2	46
36	2-Ethyl-5-isobutylthiophene	250	−25	−180
37	3,3,6,6-Tetramethyl-1,2,5-trithiepane	500	−22	51
38	3,3,7,7-Tetramethyl-1,2,5-trithiepane	500	25	18
39	3,3,8,8-Tetramethyl-1,2,5,6-tetrathiocane	500	46	39
40	3,3,7,7-Tetramethyl-1,2,5,6-tetrathiocane	250	30	20
41	2-Pentylpyridine	1497	29	78
42	Furfuryl isovalerate	617	56	84
43	2-Furylmethanol	500	22	46
44	Tropone	500	29	36
	**Common Compounds**			
45	3-Methylbutanol	500	13	61
		250		14
46	2-Phenylethanol	500	45	69
		250		23
		50		31
47	(2*R*,3*R*)-Butanediol	500	−14	7
48	Acetoin	500	23	23
49	Geosmin	500	13	21
50	(+)-*R*-Methylisoborneol	250	3	69
		25		17
51	(−)-*S*-Methylisoborneol	500	12	75
		250		24
		50		10
52	Dimethyl disulfide	500	17	26
